# Gambling frequency and symptoms of attention-deficit hyperactivity disorder in relation to problem gambling among Swedish adolescents: a population-based study

**DOI:** 10.1080/03009734.2017.1294636

**Published:** 2017-04-24

**Authors:** Charlotta Hellström, Philippe Wagner, Kent W. Nilsson, Jerzy Leppert, Cecilia Åslund

**Affiliations:** a Centre for Clinical Research, Uppsala University, Västmanland County Hospital, Västerås, Sweden;; b School of Health, Care and Social Welfare, Mälardalen University, Västerås, Sweden

**Keywords:** Adolescent, attention-deficit hyperactivity disorder symptoms, gambling, problem gambling, Problem Gambling Severity Index

## Abstract

**Aim:**

To investigate the associations between gambling frequency, attention-deficit hyperactivity disorder (ADHD) symptoms, and problem gambling among adolescent boys and girls. One hypothesis was that adolescents with increased ADHD symptoms have a higher frequency of gambling compared to adolescents with fewer ADHD symptoms.

**Method:**

A population-based sample of adolescents (aged 15–18 years) completed a questionnaire on demographics, gambling habits, ADHD symptoms, and problematic gambling; 1412 adolescents (from 4440 sampled) with gambling experience were included in the final sample.

**Results:**

A zero-inflated negative binomial regression analysis revealed that increased ADHD symptoms, higher gambling frequency, and higher age were associated with lower odds for being non-susceptible to gambling problems. Moreover, gambling frequency interacted with ADHD symptoms in predicting probability of being non-susceptible to gambling problems. However, when analysing those already susceptible to problem gambling, ADHD symptoms did not modify the effect of gambling frequency on the expected magnitude of gambling problems. In susceptible individuals, problem gambling increased with both increased ADHD symptoms and increased gambling frequency, but the level of problems due to gambling frequency did not change depending on the ADHD symptom level. There was an interaction effect between sex and gambling frequency in relation to gambling problems.

**Conclusions:**

Adolescents with ADHD symptoms seem to be more sensitive to gambling, in terms of being susceptible to developing gambling problems. However, once susceptible, adolescents with ADHD symptoms are affected by gambling frequency similarly to other susceptible participants.

## Introduction

Approximately 0.2%–0.3% of the general population is diagnosed with gambling disorders (1) leading to individual suffering and major societal costs. Problematic gambling has been conceptualized, defined, and measured in different ways (2). In the present study, the terms *problematic gambling*, *problem gambling*, and *gambling problems* have been used interchangeably to describe the negative effects of gambling. Individual demographics such as sex and age are risk factors for problematic gambling (3). Among young adults, problematic gambling has been associated with negative emotions and personality issues (4). Moreover, problematic gambling behaviour has been associated with poor general health (5), and individuals diagnosed with a gambling disorder have high rates of comorbidity with other mental health disorders such as depression, anxiety, and personality disorders (1).

Attention-deficit hyperactivity disorder (ADHD) has been associated with adolescent gambling behaviours, although gambling problems seem to be more related to hyperactivity/impulsivity than to inattention (6). Faregh and Derevensky found no interactions between ADHD symptoms and gambling in relation to gambling pathology; however, ADHD subtypes and gambling severity were unequally associated with depressive affect and emotional problems (7).

Young people are more neurobiologically vulnerable compared with adults and are at higher risk of developing problematic gambling, partly because of their underdeveloped ability to predict consequences due to actions (i.e. gambling) (8). Hyperactivity, low impulse control, and inattention are important issues in research about gambling behaviour among adolescents (7). ADHD at younger ages often persists into adulthood (9). The prevalence of ADHD ranges from 2.2% to 17.8% among children and adolescents aged 10–20 years (9), whereas the prevalence of ADHD among adults is estimated at 2.5% (1). The most common reason for child and adolescent psychiatry referral is symptoms of either depression or ADHD (10). Impulse control problems, as well as a lower capability to predict consequences due to one’s actions, are common factors among adolescents with ADHD (8,11). This might further increase the risk for other problems and disorders, such as negative social relationships and mental health disorders (8,11).

Frequencies of different ADHD symptoms differ between boys and girls. For example, externalizing disorders such as hyperactivity and impulsive behaviours are more common among boys with ADHD; girls with ADHD more often show internalizing disorders including inattention (12). Internalizing disorders may be more difficult to identify than hyperactivity. Furthermore, inattention has been suggested as the most common ADHD subtype (7). Since girls with ADHD have more inattention problems, there is a significant possibility that ADHD among girls remains underdiagnosed (13). Young adults who reported earlier childhood ADHD symptoms experience more gambling-related problems than are found in the general population (14). However, other studies report contradictory results (15). Nevertheless, impulsivity is inherent in both problematic gambling and ADHD (16), in which the hyperactivity and impulsivity subtypes are more closely related to gambling than is the inattention subtype (7). Individuals with hyperactive behaviour prefer quicker, smaller rewards instead of larger rewards that take longer to achieve (17). These impulsive ‘quick win’ preferences are also present among problem gamblers (18). With the growing interest in adolescents’ gambling habits, there is a need for greater knowledge about ADHD symptoms among adolescent gamblers (6).

The study aim was to investigate the associations between gambling frequency, ADHD symptoms, and problematic gambling among adolescent boys and girls. Our hypothesis was that the additive effect of increased gambling frequency and increased ADHD symptoms would be associated with increased gambling problems.

## Materials and methods

### Participants and procedures

**Figure 1.**
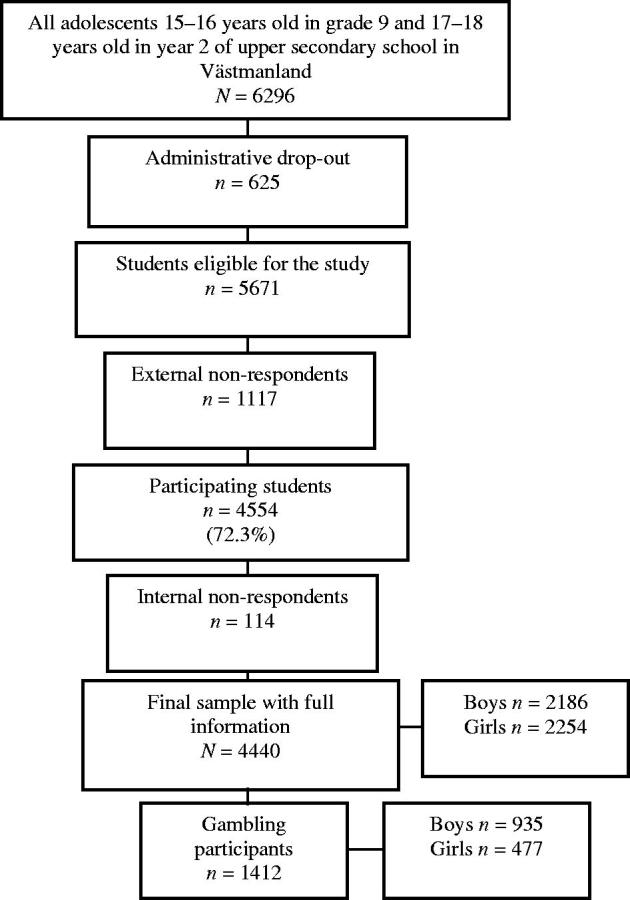
Flow chart of the study population. Administrative drop-out refers to students from classes or schools that did not participate in SALVe 2012. External non-respondents are students who were absent on the day of data collection and did not return their questionnaire by mail or who declined to participate. Those who did not sufficiently complete the questions for the present study were referred to as ‘internal non-respondents’.

This study was part of SALVe 2012, a survey distributed biennially by the Swedish County Council of Västmanland to monitor the health and life situation of the county’s adolescent population. The adolescent study population is from a county considered fairly representative of Swedish society because of its distribution of educational, income, and employment levels, as well as its urban and rural areas (19). The survey includes demographics, ADHD symptoms, and gambling habits and problematic gambling items. All students in the 9th grade (15–16-year-olds) of compulsory school and the second year of upper secondary school (17–18-year-olds) in Västmanland were the target population. The students answered the questionnaire during class (administered by their teacher) and were informed that participation was voluntary, anonymous, and that they could end their participation at any time. Non-attending students were given a second chance to complete the questionnaire and were defined as late-respondents. The final sample was 4440 adolescents (2186, 49.2% boys; 126 late-respondents). In the final statistical models, only adolescents with gambling experience were included (*n* = 1412). A flow chart of the study population is presented in Figure 1.

### Ethical considerations

The study followed the Swedish guidelines for studies of social science and humanities according to the Declaration of Helsinki. According to Swedish law (Ethical Review Act 2003:460), this type of anonymous study is not subject to ethical approval.

### Measures

*Sex.* Participants were coded as boy (code 1) or girl (code 2).

*Parents’ country of birth.* Participants with both parents born in Sweden or Scandinavia were classified as Scandinavian ethnicity (code 1); those with at least one parent born outside Scandinavia were classified as non-Scandinavian ethnicity (code 2).

*Family constellation.* Parents were coded based on living together (code 1) or not living together (code 2).

*Parental employment status.* Parents were coded as ‘both employed’ (code 1) or ‘one or both unemployed’ (code 2).

*Subjective socio-economic status (SES).* SES was reported on a 7-point Likert scale (20), where participants were asked to rank the SES of their family; steps 1–3 indicated the lowest status, steps 4–5 was categorized as medium status, and step 7 indicated the highest status. Participants were asked to imagine that society could be understood as a ladder where people at the lowest steps have the least money and those at the highest top are the ones with a lot of money. By referring to how wealthy they thought their own family was when compared to the rest of the society, they were asked to place their family on the scale.

*ADHD symptoms.* The World Health Organization Adult ADHD Self Report Scale (ASRS) (21) was used. Of the 18 ASRS questions, the first six are valid for use alone as a short screening (ASRS-S) (22,23), which was used here. Participants were asked about their frequency of ADHD symptoms within the last 6 months, with response options: never (0 points), rarely (1 point), sometimes (2 points), often (3 points), and very often (4 points); giving a range of 0–24 points. The ASRS-S was validated in an adult non-clinical sample with sensitivity 68.7% and specificity 99.5% (21). To create a dichotomized ADHD symptom variable, the cut-off score of ≥4 was used (21). Scores of 0–3 were categorized as no ADHD (code 0); 4 or more were categorized as ADHD (code 1).

*Attention deficit and hyperactivity subgroups.* The ASRS-S has two latent factors, attention deficit and hyperactivity (24), that were investigated as separate summation indices in subgroup analyses. Internal consistency values were 0.79–0.87 for the inattention subscale and 0.68–0.89 for the hyperactivity subscale (23). A dichotomous cut-off point was set for each item (21).

*Gambling frequency*. Items included: Online gambling frequency (‘How often do you gamble on poker, casino or similar on the Internet for real money?’); Offline poker gambling frequency (‘How often do you play poker for money (not online)?’); Gambling frequency on the lottery or other games (‘How often do you gamble for money on the lottery or other games (horse-racing, scratch cards, sports, etc., not online)?’); and Gambling frequency on slot machines (‘How often do you gamble on slot machines (the sort you can win money on, not online)?’).

Response options for each gambling frequency question were: never (0 points), a few times a year (1 point), once a month (2 points), 2–4 times a month (3 points), 2–3 days a week (4 points), 4–5 days a week (5 points), and 6–7 days a week (6 points) (Table 1). We examined the associations between the four gambling frequency variables to identify components. Factor analysis (varimax with Kaiser normalization) revealed one component with an eigenvalue over 1.0 (2.497), factor loading = 0.763–0.853. Cumulative variance was 62.414%. Finally, the *gambling frequency index* was created by summing the factor scores for each item.

**Table 1.** Descriptive statistics for the sample of adolescents aged 15–18 years.

	Total	Boys	Girls		
	*n* (%)	*n* (%)	*n* (%)	Chi-square	*p*
Age					
15–16 years	2011 (45.3%)	997 (45.6%)	1014 (45.0%)		
17–18 years	2429 (57.7%)	1189 (54.4%)	1240 (55.0%)	0.173	0.677
Ethnicity					
Scandinavian	3434 (78.6%)	1654 (77.3%)	1780 (79.8%)		
Non-Scandinavian	937 (21.4%)	487 (22.7%)	450 (20.2%)	4.274	0.039
Parental living condition					
Parents living together	2802 (63.3%)	1413 (65.0%)	1389 (61.7%)		
Parents not living together	1622 (36.7%)	761 (35.0%)	861 (38.3%)	5.067	0.024
Parental working status					
Both parents employed	3582 (81.4%)	1774 (82.1%)	1808 (80.6%)		
At least one parent unemployed	821 (18.6%)	387 (17.9%)	434 (19.4%)	1.524	0.217
Symptoms of ADHD					
No symptoms of ADHD	3605 (81.2%)	1819 (83.2%)	1786 (79.2%)		
Symptoms of ADHD	835 (18.8%)	367 (16.8%)	468 (20.8%)	11.480	<0.001
Online gambling frequency					
Never	3834 (90.2%)	1714 (82.6%)	2120 (97.5%)		
A few times a year	164 (3.9%)	138 (6.7%)	26 (1.2%)		
Once a month	88 (2.1%)	77 (3.7%)	11 (0.5%)		
2–4 times a month	76 (1.8%)	65 (3.1%)	11 (0.5%)		
2–3 days a week	31 (0.7%)	28 (1.4%)	3 (0.1%)		
4–5 days a week	21 (0.5%)	20 (1.0%)	1 (0.0%)		
6–7 days a week	35 (0.8%)	32 (1.5%)	3 (0.5%)	266.480	<0.001
Offline poker gambling frequency					
Never	3887 (89.2%)	1734 (81.4%)	2153 (96.6%)		
A few times a year	305 (7.0%)	248 (11.6%)	57 (2.6%)		
Once a month	84 (1.9%)	77 (3.6%)	7 (0.3%)		
2–4 times a month	44 (1.0%)	36 (1.7%)	8 (0.4%)		
2–3 days a week	20 (0.5%)	17 (0.8%)	3 (0.1%)		
4–5 days a week	7 (0.2%)	7 (0.3%)	0 (0.0%)		
6–7 days a week	12 (0.3%)	12 (0.6%)	0 (0.0%)	267.702	<0.001
Offline gambling frequency on the lottery or other games					
Never	3159 (72.4%)	1459 (68.4%)	1700 (76.1%)		
A few times a year	789 (18.1%)	348 (16.3%)	441 (19.7%)		
Once a month	207 (4.7%)	141 (6.6%)	66 (3.0%)		
2–4 times a month	105 (2.4%)	89 (4.2%)	16 (0.7%)		
2–3 days a week	70 (1.6%)	62 (2.9%)	8 (0.4%)		
4–5 days a week	19 (0.4%)	17 (0.8%)	2 (0.1%)		
6–7 days a week	17 (0.4%)	17 (0.8%)	0 (0.0%)	175.575	<0.001
Offline gambling frequency on slot machines					
Never	3664 (84.1%)	1677 (78.8%)	1987 (89.1%)		
A few times a year	554 (12.7%)	331 (15.6%)	223 (10.0%)		
Once a month	67 (1.5%)	56 (2.6%)	11 (0.5%)		
2–4 times a month	31 (0.7%)	28 (90.3%)	3 (0.1%)		
2–3 days a week	17 (0.4%)	13 (1.3%)	4 (0.2%)		
4–5 days a week	9 (0.2%)	8 (0.4%)	1 (0.0%)		
6–7 days a week	14 (0.3%)	14 (0.7%)	0 (0.0%)	119.554	<0.001
Problem Gambling Severity Index					
No risk/low risk of problem gambling (0–2 points)	1222 (86.5%)	763 (81.6%)	459 (96.2%)		
Moderate risk of problem gambling (3–7 points)	117 (8.3%)	106 (11.3%)	11 (2.3%)		
High risk of problem gambling (8 points)	73 (5.2%)	66 (7.1%)	7 (1.5%)	57.992	<0.001

*Problem gambling.* Only participants identified as gamblers completed the Problem Gambling Severity Index (PGSI) (25), nine questions on gambling behaviour used for measuring gambling problem severity (range 0–27 points). The PGSI was originally categorized into four levels separating non-problem gambling and low risk for problem gambling (26). For all analyses, we used the PGSI summation index as a continuous variable (25). With the exception of the descriptive analyses (Table 1), the PGSI was categorized into three levels with the first two combined: 1) non-problem gambling and low risk for problem gambling (0–2 points); 2) moderate risk for problem gambling (3–7 points); and 3) high risk for problem gambling (8 points). PGSI internal consistency (Cronbach’s α) was 0.92.

### Statistical analyses

Fisher’s exact probability test was used to investigate whether the gambling frequency index, ADHD symptoms, and the PGSI differed between late-respondents and other participants. Sex differences were analysed using Pearson’s chi-square and Mann–Whitney *U* tests. Factor analysis (varimax with Kaiser normalization) was used to examine associations between gambling frequency and to identify components or higher-order groups of gambling frequency within different types of gambling forms. Kendall’s Tau and Cramer’s V were used to investigate correlations between study variables of gambling frequency (*online gambling frequency*, *offline poker gambling frequency*, *gambling frequency on the lottery or other games*, *gambling frequency on slot machines*), ADHD symptoms, and PGSI.

Because most study participants did not have gambling problems according to the PGSI, and because a few cases were quite severe, PGSI data were significantly skewed. The magnitude of skew was such that the analyses could not be conducted using ordinary analysis methods. Instead, because the skew was partly caused by multiple zero-order measurements, zero-inflated negative binomial regression was used. This method combines and simultaneously estimates two separate regression models: the first is a logistic regression model accounting for the excess zero measurements; the second is a negative binomial regression model used to analyse the expected PGSI measurements. *Model I* accounts for the excess zeros by modelling the probability of being non-susceptible to gambling problems. It demands a non-event as the outcome because the main interest is in the excess of zeros. The outcome of being non-susceptible to gambling problems is therefore in contrast to the standard logistic regression that would model the risk of having gambling problems. The subsequent results are interpreted as the odds ratio (OR) of being non-susceptible to developing gambling problems. The model included covariates: ADHD symptoms, gambling frequency index, a gambling frequency index by sex interaction, and a gambling frequency index by ADHD symptom interaction (the last-mentioned was of primary interest).

*Model II* used negative binomial regression to analyse the magnitude of gambling problems in susceptible individuals. Resulting estimates were interpreted as the ratio of expected PGSI points, given that the participant is susceptible, and included the same covariates. The same statistical analyses were performed on the ADHD symptoms subgroups hyperactivity and inattention. All analyses were controlled for potential confounding demographic variables (age, sex, parents’ country of birth, parental working status, parental living condition, and SES). As part of the modelling process, to maximize statistical power, covariates and factors that were not statistically significant and that did not alter the gambling by ADHD interaction estimate, whether included or removed, were removed from the model.

The level for statistical significance was set at *p* < 0.05. Analyses were performed using IBM SPSS statistics, version 22 (IBM Corporation, Armonk, NY) and STATA version 12 (StataCorp, College Station, TX).

## Results

Roughly 10% of adolescents gambled on online poker/casino and offline poker; gambling on slot machines was slightly more common (Table 1). Nearly one in three adolescents reported gambling on lotteries, horse-racing, scratch cards, and sports. Compared with girls, boys had higher frequencies of gambling in every form. Online poker and/or casino were most common among weekly gamblers.

About one-third (*n* = 1412) of the sample reported gambling according to the PGSI; 117 participants fulfilled the criterion for moderate risk of problem gambling and 73 for problem gambling. The mean PGSI was markedly higher among boys (*M* = 1.64, SD = 3.68) than girls (*M* = 0.40, SD = 1.80, *Z* = −9.007, *p* < 0.001) (data not shown). Late-respondents did not differ on the main variables of gambling frequency, ADHD symptoms, and PGSI (data not shown). Most participants were of Scandinavian ethnicity and lived with both parents, who were predominantly employed (Table 1).

Mean SES was marginally higher among boys (*M* = 4.56, SD = 1.02) than girls (*M* = 4.37, SD = 0.97, *Z* = −5.867, *p* < 0.001). About one in four participants reported ADHD symptoms (Table 1). Mean ADHD symptoms among boys (*M* = 8.52, SD = 4.73) was lower than among girls (*M* = 9.42, SD = 4.73, *Z* = −6.128, *p* < 0.001).

Table 2 shows the correlation coefficients between gambling frequency, ADHD symptoms, and PGSI. Correlations were found for all gambling forms, indicating that gamblers often use several methods. All gambling forms were moderately correlated with PGSI; ADHD symptoms were weakly correlated with PGSI. Although statistically significant, the ADHD symptoms index was only negligibly correlated with the different gambling forms.

**Table 2.** Kendall’s Tau (and Cramer’s V for sex) correlations between study variables.

		Total population (*n* = 4440)					Gambling population (*n* = 1412)	
Factors	1	2	3	4	5	6	7	8
1. Sex	1	0.144***	0.250***	0.248***	0.201***	0.166***	0.353***	0.250***
2. Symptoms of ADHD		1	0.040**	0.043**	0.035**	0.065**	0.050**	0.134**
3. Online gambling frequency			1	0.508**	0.310**	0.302**	0.503**	0.417**
4. Offline poker gambling frequency				1	0.351**	0.365**	0.534**	0.424**
5. Offline gambling frequency on the lottery or other games					1	0.334**	0.721**	0.341**
6. Offline gambling frequency on slot machines						1	0.592**	0.369**
7. Gambling frequency index							1	0.431**
8. Problem gambling								1

** *p* < 0.01 level (2-tailed); *** *p* < 0.001 level (2-tailed).

The ORs in Table 3 for Model I show the probability of being non-susceptible to developing gambling problems since the model demanded a non-event as the outcome. The interpretation of the logistic regression in Model I is therefore that an OR above 1.0 indicates an increased probability of being non-susceptible to gambling problems and an OR below 1.0 indicates a decreased probability of being non-susceptible to gambling problems (i.e. an increased probability of being susceptible). As part of the modelling process, covariates that were not statistically significant and that did not alter the gambling by ADHD interaction estimate were removed. An increase in ADHD symptoms was associated with lower probability of being non-susceptible to gambling problems (Table 3, Model I). Higher gambling frequency and higher age were associated with lower probability of being non-susceptible to gambling problems (Table 3, Model I). A statistically significant interaction was observed between ADHD symptoms and the gambling frequency index when studying the probability of being non-susceptible for developing gambling problems (Table 3, Model I).

**Table 3.** Zero-inflated negative binomial regression analysis, adjusted for an excess of zero values for associations between investigated variables; dependent variable: PGSI.

Model I. Analysis of the chance of not becoming susceptible to developing gambling problems.^a^				
	OR	*p*	95% CI	
Age	0.48	0.001	0.30	0.75
ADHD symptoms index	0.95	0.041	0.90	1.00
Gambling frequency index	0.46	0.009	0.26	0.82
ADHD symptoms × Gambling frequency index	0.92	0.023	0.85	0.99
Model II. Analysis of adolescents already susceptible to problem gambling.^b^				
	IRR	*p*	95% CI	
ADHD symptoms index	1.03	0.031	1.003	1.07
Gambling frequency index	1.34	<0.001	1.20	1.51
Sex (ref: boys)	0.25	<0.001	0.16	0.38
Sex × Gambling frequency index	1.67	<0.001	1.34	2.13

a Model I: adjusted for covariates sex and parents’ country of birth.

b Model II: adjusted for covariate age.

In Table 3, Model II, the zero-inflated negative binomial regression revealed that an increase in gambling frequency index and ADHD symptoms was related to higher PGSI scores among individuals susceptible to problem gambling. Susceptible girls were less likely to develop gambling problems than susceptible boys, and a significant interaction effect was revealed between sex and gambling frequency in relation to gambling problems (*p* < 0.001).

**Figure 2.**
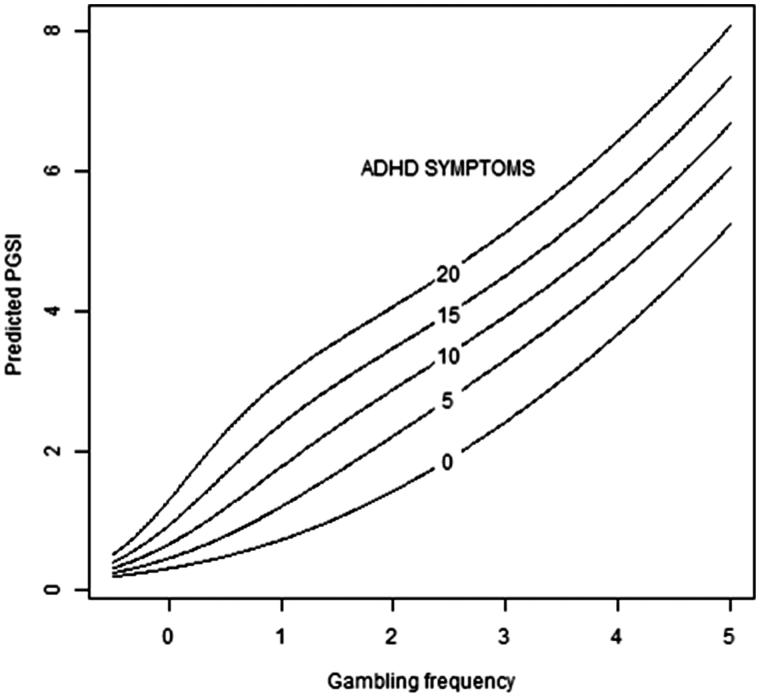
Illustration of the association between gambling frequency and the Problem Gambling Severity Index (PGSI). The expected degree of gambling problems in the study sample, irrespective of susceptibility to gambling problems, plotted against gambling frequency for different degrees of ADHD symptoms according to the ADHD symptom index.

The results in Table 3, Model I, show that the effect of gambling frequency index on becoming susceptible to gambling problems increases with increasing ADHD symptoms. The magnitude of these differences is further illustrated in Figure 2, which shows that increased ADHD symptoms and increased gambling frequency were associated with increased PGSI scores, although causality was not investigated.

Furthermore, when analysing the probability of being non-susceptible to developing gambling problems within the ADHD subgroups, neither hyperactivity symptoms nor inattention symptoms revealed significant interaction effects between the degree of hyperactivity/inattention symptoms and gambling frequency index (*Z = *−0.37, *p = *0.711 and *Z = *−0.70, *p = *0.482) (data not shown).

However, the zero-inflated negative binomial regression used to analyse ADHD subgroups revealed significant interaction effects between the degree of hyperactivity symptoms and gambling frequency index in relation to PGSI scores among individuals susceptible to problem gambling (Z = −2.23, *p = *0.026), while no significant interaction effect between inattention symptoms and gambling frequency was found (Z = −1.86, *p = *0.063) (data not shown).

## Discussion

This study explored the associations between gambling frequency, ADHD symptoms, and PGSI among Swedish adolescents. Adolescents with ADHD may be more sensitive to gambling frequency, in terms of becoming susceptible to developing a gambling problem. However, once susceptible, adolescents with ADHD symptoms are equally affected compared with other susceptible participants based on gambling frequency. Furthermore, gambling frequency seems to be of greater importance for problem gambling than ADHD symptoms among adolescents.

In previous research, the associations between gambling frequency, gambling problems, and ADHD symptoms have been reported with varying results (6,7,27), causing debate about possible causality. In the present study, gambling frequency and ADHD symptoms interacted in relation to the probability of being non-susceptible to gambling problems. However, in individuals already susceptible to gambling problems, the association between gambling frequency and gambling problems did not change depending on the ADHD symptom level.

Notably, girls already susceptible to gambling problems had lower odds for developing gambling problems than did boys. Furthermore, interaction effects were revealed between sex and gambling frequency regarding problem gambling, indicating that sex may not be a simple determining PGSI factor but also an influencing factor for gambling frequency. This finding contributes to previous research showing sex to be a primary risk factor for developing problem gambling behaviours (3). Since adolescents are more neurologically vulnerable than adults to developing addictions (8), it would be of further interest to investigate whether boys and girls differ within those neurobiological factors associated with how reward system neural circuits contribute to the development of gambling problems. The higher prevalence of ADHD among girls may be partly explained by our use of the ASRS-S, for which respondents self-report ADHD symptoms. In previous research (13), girls’ ADHD symptoms were often undetected, in part because girls with ADHD have more inattention problems than do boys with ADHD, who are more hyperactive. Nevertheless, the ASRS-S has been validated and is considered a reliable screening instrument (23). Since hyperactivity symptoms interacted with the gambling frequency index for those already susceptible to gambling problems, and inattention symptoms did not (although those differences were minor), we suggest that further research should assess interaction effects within ADHD subgroups as well as psychological and social differences between boys and girls (12).

It should be mentioned that data from SALVe 2012 have previously been analysed in Hellström et al. (28). Regardless of different aims and included study variables, there are obviously similarities due to study planning, study population, and methods described in the present study.

Several limitations of the study should be noted. First, because all analyses were based on self-reports, there is a risk of information bias due to false or inaccurate answers or misunderstanding the questions. Second, since the design was cross-sectional, we could only speculate on the possible direction of associations because there was no way to predict causality. Third, although gambling activity is not exclusive to adolescents, our sample was limited to this population; the results should not necessarily be considered representative of other age groups. Fourth, gambling activities are more common among boys (29), whereas girls more often suffer from mental health issues (22). The influences of undetected ADHD symptoms among girls should be noted since inaccurate measures for detecting ADHD symptoms in girls may mean that prevalence among this group is underestimated (22).

Fifth, we investigated only ADHD symptoms, not clinical diagnosis. However, self-reported screening for ADHD symptoms may be an effective complement for identifying adolescents at psychiatric risk (22). ASRS-S is seen as an accurate and reliable method for this purpose (23). Our ability to find interaction effects was limited due to low power, because few adolescents in our sample reported gambling problems. This may be partly because gambling under 18 years of age is illegal in Sweden. The fact that only about one-third of the adolescents gambled may have influenced our results. That said, the statistical method of zero-inflated negative binomial regression was specifically chosen to adjust for the skew in the study population caused by an excess of zero measurements on the PGSI. Further, adolescents with gambling problems may have participated in the study to a lesser extent. It is possible that they prefer to gamble instead of participate in school activities and may have been absent from school on the day of data collection. However, late-respondents did not differ from other participants on any of the dependent or independent factors; non-respondents tend to be similar to late-respondents on survey studies (30).

We were unable to investigate further age differences, and age was instead included as a covariate in all analyses. Future research on the associations between ADHD and gambling problems should consider possible age effects.

How well the PGSI separates low-frequency gamblers from medium-frequency gamblers has been the subject of debate (25). Because the present study only used these categorical variables as descriptive measures and the continuous PGSI summation index for all other analyses, the risk of underestimating effects is more likely than the risk of overestimating them. Although analyses were controlled for potential confounding variables, there is the risk that other confounding factors that were not adjusted for may have interfered.

The present study also has several strengths. To our knowledge, this is the first study to investigate the associations between the frequency of different gambling forms and ADHD symptoms in relation to problem gambling among adolescents. Another strength is the large community adolescent sample from a county that may be considered to represent Sweden as a whole (19). The rather high participation rate reduces the risk of selection bias. The results may thus generalize to adolescent populations from other countries with similar cultures and living conditions.

The exploratory design of the present study contributes to the growing literature on associations between problematic gambling behaviour and ADHD. Adolescents with ADHD symptoms seem to be more sensitive to gambling in terms of becoming susceptible for developing gambling problems. However, once susceptible, adolescents with ADHD symptoms are affected similarly to other susceptible participants based on gambling frequency. Interaction effects between the frequency of gambling and ADHD subgroups (hyperactivity and inattention) further suggest that different personality traits are associated to problem gambling. Information about factors related to gambling problems may be of particular interest to mental health care, psychiatry, psychology, and social work clinicians, as well as policy-makers, parents, and teachers involved in adolescent health and development.

Consistent with previous research, the co-occurrence of gambling frequency and ADHD symptoms in relation to problematic gambling further emphasizes the importance of co-screening for both gambling and ADHD symptoms when examining problematic gambling among adolescents (31). However, replication of the results and identification of the direction of causality are needed before interventions should be developed. Moreover, further investigation of gambling frequency and ADHD symptoms in relation to problem gambling in clinical settings is needed.
